# Building nanogapped graphene electrode arrays by electroburning†

**DOI:** 10.1039/c7ra13106b

**Published:** 2018-02-12

**Authors:** Chunhui Gu, Dingkai Su, Chuancheng Jia, Shizhao Ren, Xuefeng Guo

**Affiliations:** Beijing National Laboratory for Molecular Sciences, State Key Laboratory for Structural Chemistry of Unstable and Stable Species, College of Chemistry and Molecular Engineering, Peking University Beijing 100871 P. R. China guoxf@pku.edu.cn; Department of Materials Science and Engineering, College of Engineering, Peking University Beijing 100871 P. R. China

## Abstract

Carbon nanoelectrodes with nanogap are reliable platforms for achieving ultra-small electronic devices. One of the main challenges in fabricating nanogapped carbon electrodes is precise control of the gap size. Herein, we put forward an electroburning approach for controllable fabrication of graphene nanoelectrodes from preprocessed nanoconstriction arrays. The electroburning behavior was investigated in detail, which revealed a dependence on the size of nanoconstriction units. The electroburnt nanoscale electrodes showed the capacity to build molecular devices. The methodology and mechanism presented in this study provide significant guidance for the fabrication of proper graphene and other carbon nanoelectrodes.

## Abstract 2

An approach for the efficient fabrication of graphene nanoelectrodes through the combination of dash-line lithography and electroburning is demonstrated in detail.

## Graphical abstract

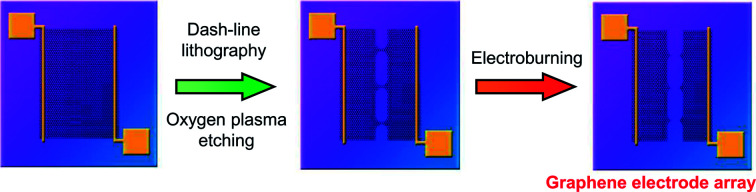

## Introduction

Nanogapped carbon electrodes are pairs of extremely close carbon materials such as carbon nanotubes or graphene. Because of their advantages including easy chemical processing, strong organic material affinity, low atomic mobility, high carrier mobility, and low screening of the gate electric fields, nanogapped carbon electrodes have attracted extensive interest and have been widely applied in molecular electronics^1–5^ and miniaturized organic transistors.^6–8^ One of the main challenges in the fabrication of nanogapped carbon electrodes is precise control of the size of the nanogap under the limited pattern resolution provided by the typical nanofabrication technologies. For instance, electron-beam (e-beam) lithography, as a principle patterning method, can achieve a resolution of sub-50 nm and even sub-10 nm with the aid of specific resists or equipment,^9,10^ which is, however, still far from the atomic scale. Therefore, the key point of a fabrication strategy is to produce a target nanostructure under patterning precision. In this regard, different kinds of methodologies, including ion/electron beam etching,^11,12^ selective plasma etching,^1,2^ and electroburning,^3,13,14^ have been developed to fabricate nanogapped carbon electrodes. Among them, electroburning is a widely applied method with the advantage of simplicity, controllability, and a lack of dependence on special equipment. In addition, electroburning benefits from the extra capability to control the formation of uncut graphene nanoconstrictions or cracked nanoelectrodes by controlling the burning degree. Hereinto, the former has been applied in the investigation of quantum effects^15–17^ and the fabrication of functionalized devices,^18–20^ whereas the latter acts as source/drain electrodes for molecular electronic devices, as discussed above.

**Fig. 1:**
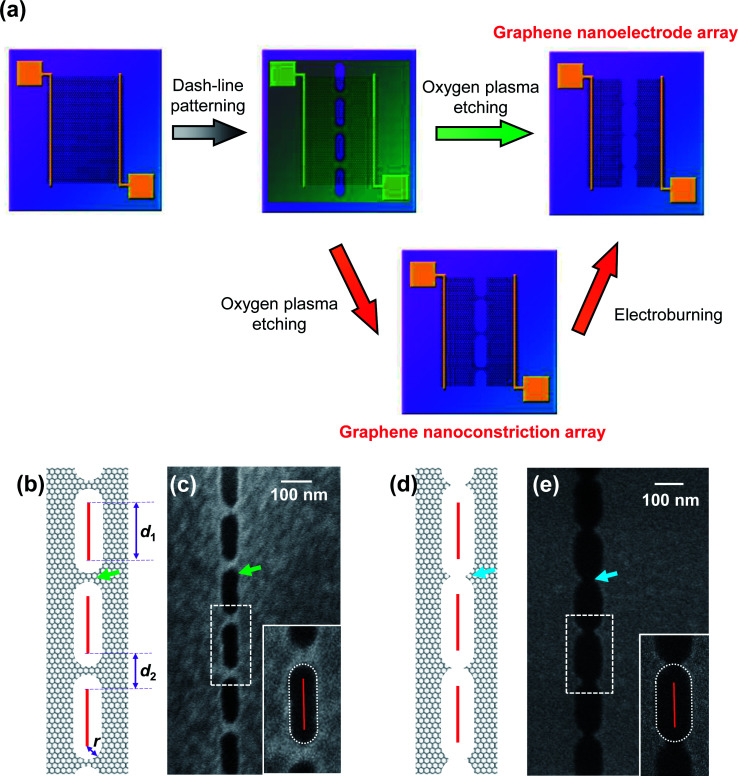
(a) Schematic of the fabrication of graphene nanoelectrode arrays. The green and red arrows show the direct cutting and electroburning routes, respectively. (b–e) Schematic and SEM images of graphene nanoconstriction arrays (b and c) and nanogap arrays (d and e) prepared by dash-line lithography. The green and blue arrows point out nanoconstriction and nanogap units. Red segments in (b and d) show the design of the e-beam pattern. The enlarged scales in (c and e) show the shape of the etching holes. The red solid segment and white dot lines show the fitted e-beam pattern and broadening range, respectively.

In our previous study, we have developed graphene nanoelectrode arrays formed through a dash-line lithographic method to fabricate single-molecule junctions (SMJs).^2^ Compared to a single unit, they have more potential binding sites and a higher possibility in an array to find a unit with a suitable interval for particular molecules that is beneficial for molecular bridging. This method is described in detail in the Experimental section as well as in literature (Fig. 1a).^2^ In general, high-quality single-layer graphene grown by a chemical vapor deposition (CVD) process was transferred to silicon wafers with a layer of thermally-grown 300 nm SiO_2_. Then, graphene was tailored into a 40 μm wide ribbon by a standard photolithographic and selective oxygen reactive ion etching (RIE) method. Metallic electrodes (Cr 8 nm + Au 60 nm) were further thermally evaporated on graphene as source and drain electrodes. A 5 nm wide dash line (Fig. 1b) with alternating segments (*d*_1_) and spaces (*d*_2_) was patterned on a polymethylmethacrylate (PMMA) mask by a standard e-beam lithographic process. Oxygen RIE was applied to selectively etch graphene from the dash-line windows. The shape of the etching holes (Fig. 1c and e inset) indicates that this pattern is derived from isotropous broadening from the pre-designed e-beam pattern with a broadening radius (*r*), which mainly results from the radius of the electron beam, the corrosion of resist development, and the etching of RIE. It is worth noting that for each unit, graphene is processed into a nanoconstriction if 2*r* < *d*_2_ (Fig. 1b and c) and a graphene nanogap if 2*r* > *d*_2_ (Fig. 1d and e). The latter can be directly applied as graphene nanoelectrodes by carefully controlling 2*r* to be slightly larger than *d*_2_. As a complement, the former can be regarded as a potential precursor to build similar arrays through an electroburning process (Fig. 1a). In this regard, the electroburning process of graphene nanoconstriction arrays is a key point to be investigated.

## Experimental

### 1. Graphene growth

Herein, 25 μm thick copper films (99.8% Alfa Aesar) were immersed in acetic acid for 30 min at room temperature to remove copper oxide and other impurities. High-quality single-layer graphene was grown on copper films by a chemical vapor deposition (CVD) method. In this procedure, the furnace was heated to 1050 °C in a 14 cm min^−1^ flow of H_2_ at a pressure of 75 Pa. After about 1 h, 1.4 cm min^−1^ of CH_4_ was introduced for 15 min, and the flow of H_2_ was adjusted to 7 cm min^−1^ at the same time. The furnace was then cooled down to room temperature.

### 2. Fabrication of graphene devices

After spin coating (4000 rpm, 30 s) of the polymethylmethacrylate (PMMA) supporting polymer (MicroChem, 495 PMMA, A6), the PMMA–graphene–copper layers were put into a solution of ferric chloride to etch copper substrates. The PMMA–graphene layers were rinsed with abundant ultrapure water and transferred to a 300 nm thick SiO_2_/Si substrate; subsequently, PMMA was totally removed by boiling acetone. A graphene sheet (40 × 2200 μm) was fabricated by the combination of photolithography and selective oxygen reactive ion etching (RIE) (15 Pa, 50 W). Finally, high-density patterned metallic electrodes (Cr 8 nm/Au 60 nm) were deposited onto the graphene sheet through photolithography and thermal evaporation.

### 3. Fabrication of graphene nanoelectrode arrays

After spin coating (4000 rpm, 45 s) with a PMMA layer (MicroChem, 950 PMMA, A5), we ran a DesignCAD file with a 5 nm-width dash line between each pair of electrodes to obtain 50 μm-length windows by e-beam lithography. The development of resist was performed in a mixture of ultrapure water/isopropanol (1 : 3) at 5 °C with the aid of ultrasonication. Considering the routine condition (*d*_1_ = 150 nm and *d*_2_ = 40 nm in the dash line; 40 μm width of graphene sheets), the number of graphene nanoconstriction units in one array was calculated to be ∼210 (Section 2, ESI†). The graphene exposed from the window was then etched by oxygen RIE (15 Pa, 50 W). The resistance of graphene was monitored to control the optimal RIE condition. After RIE, the PMMA layer was totally removed by boiling acetone. Electroburning was carried out using an Agilent 4155C semiconductor characterization system. A slow voltage ramp (100 mV s^−1^) was applied to the device until the current dropped in magnitude to ∼pA.

### 4. Fabrication of single-molecule junctions

The devices were immersed in a pyridine solution containing a mixture of amino-terminated molecules (0.1 mM) and the dehydrating/activating agent 1-ethyl-3-(3-dimethylaminopropyl) carbodiimide hydrochloride (EDCI) (1 mM) under the protection of argon. After reacting for 72 h, the devices were removed from solution, rinsed with acetone and isopropanol, and completely dried with a stream of N_2_ gas. The *I*–*V* characteristics were determined using an Agilent 4155C semiconductor characterization system.

## Results and discussion

**Fig. 2:**
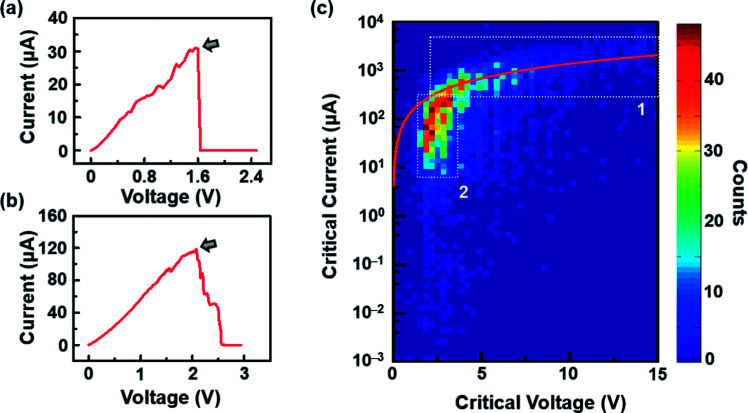
(a and b) Representative *I*–*V* characteristics in the electroburning process. The arrows indicate the critical points. (c) Two-dimensional histogram of the critical voltage against the critical current in a semi-logarithm axis. The white dot line contours show the ranges of Regions 1 and 2. The red line shows the results from a model based on a classical resistance law.

After the RIE process was conducted under optimal conditions, the resistance of graphene significantly increased (Tables S1 and S2, ESI†) due to the formation of nanoconstrictions. The electroburning process was then carried out in air at room temperature. A slow voltage ramp (100 mV s^−1^) was applied to the graphene nanoconstriction arrays, and the corresponding current was determined at 10 Samples per s (Sa per s). Fig. 2a and b show the representative current–voltage (*I*–*V*) characteristics of the electroburning process. Generally, the current increases along with the voltage up to a critical point at which electroburning is induced and the current rapidly drops to ∼pA with one (Fig. 2a) or several (Fig. 2b) steps. From 4302 samples of graphene nanoconstriction arrays, we determined the voltage and current at the critical points. Fig. 2c shows the two-dimensional histogram of the critical voltage against the critical current in a semi-logarithm axis (Fig. 2c). Without considering the transition area, the critical points are mainly distributed in two regions: the critical current region >300 μA is defined as Region 1, in which the critical current is nearly linear with the critical voltage; the area where the critical current region is 6–300 μA and the critical voltage region is 2–3 V is defined as Region 2, in which the critical voltage is nearly invariable with the critical current. This distribution may imply two electroburning regimes.

To understand this regime, we initially regard graphene as a classical two-dimensional resistor obeying the law of resistance (eqn (1)):1
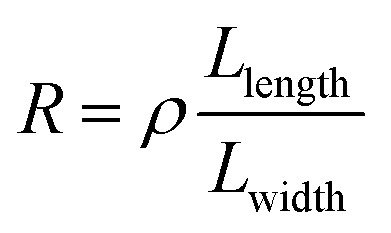
which has been proven suitable for two-dimensional materials such as large-area graphene.^21^ Herein, *R* is the resistance, *ρ* is the resistivity of graphene, which has been determined as ∼25.1 ± 8.1 kΩ for single-layer graphene (Fig. S3b, ESI†), and *L*_length_ and *L*_width_ represent the length and width of the conductive channel, respectively. We simply consider that the graphene nanoconstriction cracks at a definite critical current intensity (*J*). For SiO_2_ substrates, *J* = ∼10^8^ A cm^−2^ according to numerous results.^3,15,21,22^ A classical theoretical model was built to show the relationship between the critical current and voltage of each graphene nanoconstriction unit (Section 3, ESI†). We found that the critical current showed a near-linear relationship with the critical voltage (Fig. 2c, red line). In experiments, the results we obtained in Region 1 well fit this linear relationship. However, the classical model obviously mismatches the results in Region 2, where the critical current is under 300 μA. The inconformity to the classical Ohm's law was mostly due to the quantum size effects, involving the generation of band gaps or quantum conductance, which significantly increase the resistivity of the nanoconstriction, similar to what happens in graphene quantum dots^23^ or nanoribbons.^24,25^ In this regard, the band-gaps obviously increase the resistance of the nanoconstriction, whereas the miniaturization of the graphene nanoconstriction results in a high proportion of edge defects, which makes it more reactive in oxidation (and results in a smaller *J*).

However, these reasons still cannot explain why Region 2 covers a wide critical current range with a nearly invariable critical voltage. We think that these results may be due to the various unit numbers instead of the intrinsic property of each unit in the nanoconstriction array. In general, the inhomogeneity of the dash line or broadening must be considered as the feature sizes of nanoconstriction are much lower than the resolution of e-beam lithography. Especially, when *d*_2_ is in the range of 2*r*, the units that 2*r* > *d*_2_ are cracked by RIE, leaving the rest units as uncut nanoconstrictions. This inhomogeneity was proved in regular SEM characterization (Fig. S2, ESI†). Thus, it is reasonable to consider that the critical current is in proportion to the number of units at an invariable critical voltage due to the parallel structure. Specially, we observed that the critical current range of Region 2 was nearly ∼50 times. In view of the unit number in one array (∼210), these results indicate that Region 2 covers the conditions from little to full proportion of uncut units in one array after RIE.

**Fig. 3:**
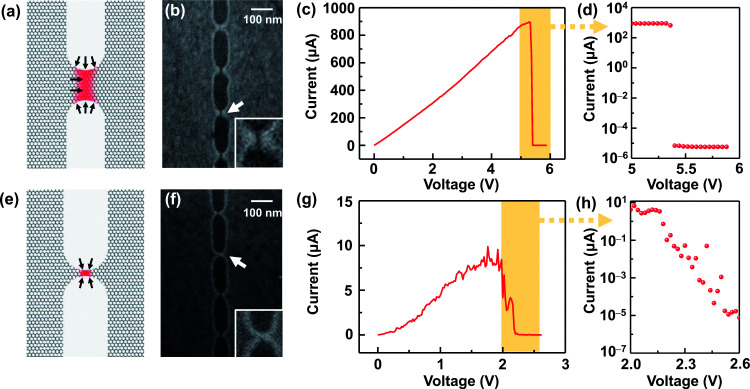
(a and e) Schematic of a nanoconstriction unit in Regions 1 (a) and 2 (e), respectively. The red area shows the hot zone. The black arrows show the probable oxidation reactive sites. (b and f) SEM images for the representative Devices 1 (b) and 2 (f) after an electroburning process, which represent Regions 1 and 2. The insets show the enlarged scale as indicated by the arrow. (c and g) *I*–*V* curves of an electroburning process for devices 1 (c) and 2 (g). (d and h) Enlarged views from the corresponding parts in (c and g) reprinted in a semilogarithmic axis.

The *I*–*V* properties may effectively reflect the electroburning process. Fig. 3c and g show the *I*–*V* properties of two representative graphene nanoconstriction arrays (Devices 1 and 2, Fig. 3a, b, e and f) in Regions 1 and 2, respectively. In comparison with Region 1, the electroburning *I*–*V* curves in Region 2 were not so sharp and direct. Their electroburning processes had more fine structures. In general, the current drops rapidly after the critical point in Device 1, whereas the current drops gradually with steps in Device 2. This phenomenon may originate from the positive feedback regimes in the electroburning process, which is similar to the electromigration process in parallel metallic electrode arrays.^26^ Considering an array with *n* identical nanoconstriction units, the current in each unit (*I*_unit_) can be evaluated according to the Ohm's law:2
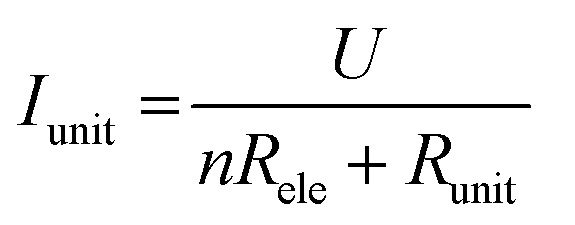
in which *U* is the applied voltage, *n* is the unit number, *R*_ele_ is the resistance of the graphene electrode, and *R*_unit_ is the resistance of each nanoconstriction unit. It is easy to find that *I*_unit_ increases when *n* decreases; this means a positive feedback: the initial breakdown of several units results in the increase of the resistance of the multiplied nanoconstriction array as well as the corresponding voltage drop, and thus, results in the current increase in each rest units, which further promotes electroburning. This regime explained why electroburning events occurred in bursts after the critical point. It is worth noting that the positive feedback ratio (*C*) equals3
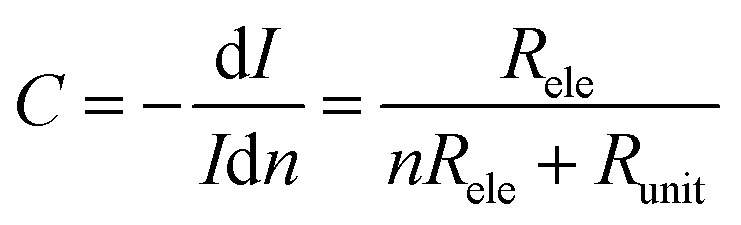
which shows that the positive feedback is much less sensitive when *R*_unit_/*n* » *R*_ele_. Experimental results indicated that the ratio between the resistance of the nanoconstriction arrays (nearly *R*_unit_/*n*) and the corresponding resistance of the electrode *R*_ele_ was from ∼0.6 to ∼12 in Region 1 and from ∼9 to ∼2500 in Region 2 (Tables S1 and S2, ESI†). This means that the voltage drop on the nanoconstriction arrays is almost identical to the applied voltage and thus invariable with the unit number in Region 2. These results may explain why the devices in Region 2 show a mild, graduated electroburning behavior.

Fig. 3d and h show the *I*–*V* properties of Devices 1 and 2 near the critical voltage in a semi-logarithm axis. The former shows a rapid current drop after the critical point, and the later shows an exponential current decay. In Regions 1 and 2, nearly ∼100% and ∼60% of devices show similar behaviors, respectively; this indicates that the electroburning process is mainly controlled by a rapid crack regime and gradual crack regime. To explain these differences, we initially accounted for the general regime of graphene electroburning. It is widely considered that electroburning is derived from the Joule heat in graphene nanoconstrictions. As a hot zone, carbon atoms in nanoconstrictions may react with oxygen in the atmosphere and then break down. Considering the generation and dispersion of Joule heat, the range of the hot zone is mainly distributed at the narrow part where the resistance is particularly large. It is worth noting that oxidation is easier to occur from the edge of graphene, which is more reactive due to the incomplete sp^2^ hybridization with abundant oxide defects.^3,12,27^ Considering the magnitude of the current in the exponential decay range (from ∼1 μA to ∼0.01 nA), we deem that this current decay derives from the electroburning process of the surviving one or several units in Device 2, which are gradually narrowed by the oxidation from the edge. However, in Device 1, the feature size of each nanoconstriction is larger. Due to the large range of the hot zone and the limited proportion of edge atoms, electroburning is unlikely to proceed only from the edges. In contrast, oxygen can react with graphene with the aid of phonons or random defects,^21,28,29^ which may cause a rapid, simultaneous oxidation in the full hot zone (and thus the rapid current drop). Specifically, the inelastic scattering of transport electrons may randomly motivate higher vibration levels, namely phonons, of chemical bonds in graphene. The higher vibration energy makes it more favourable for reaction, which allows initial oxidation inside graphene. Both electroburning regimes have been supported by previous reports.^12,28^ SEM images of Devices 1 and 2 (Fig. 3b and f) exhibit a ∼8 nm strip-typed gap and an SEM indistinguishable point-contacted gap, respectively, after the electroburning process, which supports the assumption.

It is worth noting that the critical points of ∼3% devices (127 from 4302) are located in the bottom of Region 2, where the critical voltage is 2–3 V and the critical current is <1 μA. In this area, the devices were almost open circuit at low bias voltages (<1.5 V), and then, the current sharply increased near the critical voltages (Fig. S6, ESI†). These *I*–*V* properties distinguish these devices from Region 2 in which the devices showed a nearly linear current ramp at low bias voltages. Considering the *I*–*V* shape and the magnitude of the current, this phenomenon may be attributed to electrostatic field-induced reconnection, which has been previously reported,^30^ rather than the simple electroburning process. In general, a powerful electrostatic field can tear a carbon chain from the edge of graphene as a bridge and cause the reconnection of a circuit at high voltages.

Considering the different electroburning behaviours discussed previously, a transition region should exist between two regions (instead of a transition point). In the transition region, there may be a competitive process between two regimes. There are two reasons to explain the existence of the transition region. First, the quantum size effect is gradual along with the shrinkage of the feature size; this means that the control of the quantum effect is continuous. Second, the specific properties of particular devices are not strictly identical; this may cause a broadening in statistics.

**Fig. 4:**
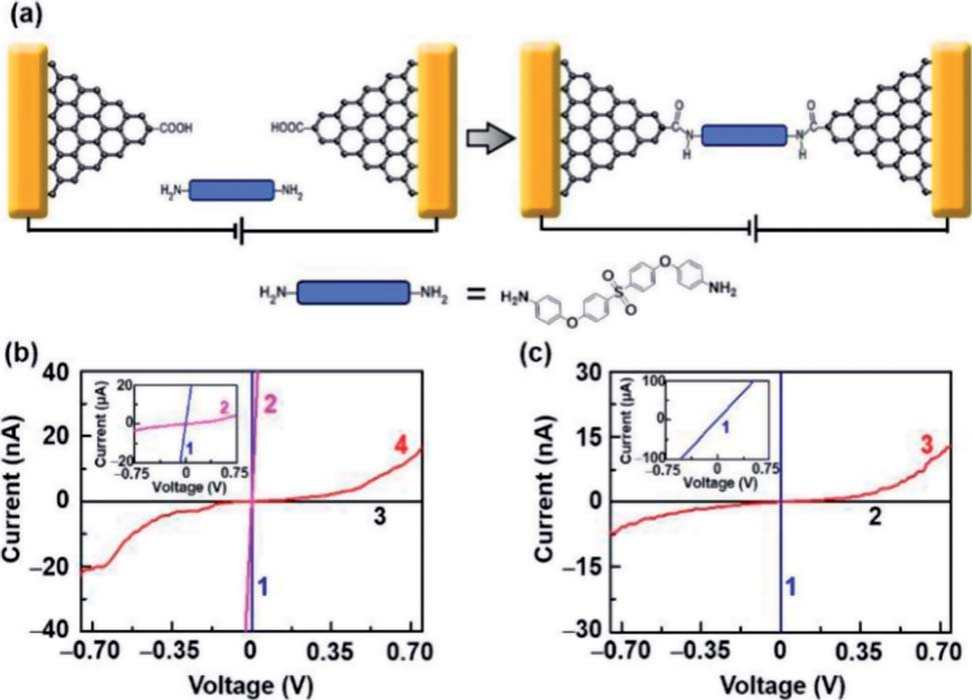
(a) Schematic of molecular immobilization on a graphene nanoelectrode unit. (b) *I*–*V* characteristics of a representative electroburnt device before oxygen etching (blue, 1), before electroburning (pink, 2), after electroburning (black, 3) and after molecular immobilization (red, 4). (c) *I*–*V* characteristics of a representative directly-cut device before oxygen etching (blue, 1), after oxygen etching (black, 2) and after molecular immobilization (red, 3), respectively.

To demonstrate the capacity of electroburnt graphene nanoelectrodes for the application, we attempted to use these devices to fabricate single-molecule junctions. In particular, amine-terminated molecules can be immobilized to nanogapped graphene electrodes, which are carboxyl functionalized at the edges due to oxidation by RIE through amide bonds (Fig. 4a, details are described in the Experimental section).^2^Fig. 4b shows the *I*–*V* characteristics of a device before oxygen etching (blue, 1), before electroburning (pink, 2), after electroburning (black, 3) and after molecular immobilization (red, 4). Especially, the current recovered to some extent after molecular immobilization, which is similar to the behavior of a direct cut device (Fig. 4c); this indicates a successful molecular connection. It is worth noting that all successful examples originate from the devices in Region 2 (linkage rate ∼27%, 14 from 52 devices in Region 2, in comparison with a linkage rate ∼0%, 0 from 89 devices in Region 1) for a small interval between the electrodes, as previously discussed. All these devices were completely confirmed and rechecked as open circuits after the electroburning process. Further, the probe voltages were set from −0.75 V to 0.75 V, which was lower than the voltage (∼1.5 V) that could induce the reconnection phenomenon. Overall, we confirmed that the current recovery resulted from the linkage of molecules rather than electrostatic field-induced reconnection.

## Conclusions

In conclusion, we developed an efficient route to fabricate nanogapped graphene electrodes in combination with dash-line lithography and electroburning. The electroburning behaviors from different graphene nanoconstriction arrays were systemically investigated, which demonstrated that the regime of electroburning depends on the feature size of nanoconstriction units. This relationship between the feature size of nanoconstriction/nanoelectrode units and the corresponding electroburning behavior is promising to offer new opportunities for future device fabrication with nanogapped electrodes.

## Conflicts of interest

The authors declare no conflicts of interest.

## Supplementary Material

RA-008-C7RA13106B-s001

## References

[cit1] Guo X., Small J. P., Klare J. E., Wang Y., Purewal M. S., Tam I. W., Hong B. H., Caldwell R., Huang L., O'Brien S., Yan J., Breslow R., Wind S. J., Hone J., Kim P., Nuckolls C. (2006). Science.

[cit2] Cao Y., Dong S., Liu S., He L., Gan L., Yu X., Steigerwald M. L., Wu X., Liu Z., Guo X. (2012). Angew. Chem., Int. Ed..

[cit3] Prins F., Barreiro A., Ruitenberg J. W., Seldenthuis J. S., Aliaga-Alcalde N., Vandersypen L. M. K., van der Zant H. S. J. (2011). Nano Lett..

[cit4] Jia C., Ma B., Xin N., Guo X. (2015). Acc. Chem. Res..

[cit5] Wei D., Liu Y., Cao L., Wang Y., Zhang H., Yu G. (2008). Nano Lett..

[cit6] Cao Y., Liu S., Shen Q., Yan K., Li P., Xu J., Yu D., Steigerwald M. L., Nuckolls C., Liu Z., Guo X. (2009). Adv. Funct. Mater..

[cit7] Qi P., Javey A., Rolandi M., Wang Q., Yenilmez E., Dai H. (2004). J. Am. Chem. Soc..

[cit8] Cao Y., Steigerwald M. L., Nuckolls C., Guo X. (2010). Adv. Mater..

[cit9] Fujita S., Maruno S., Watanabe H., Ichikawa M. (1996). Appl. Phys. Lett..

[cit10] Manfrinato V. R., Stein A., Zhang L., Nam C. Y., Yager K. G., Stach E. A., Black C. T. (2017). Nano Lett..

[cit11] Horiuchi K., Kato T., Hashii S., Hashimoto A., Sasaki T., Aoki N., Ochiai Y. (2005). Appl. Phys. Lett..

[cit12] Jin C., Lan H., Peng L., Suenaga K., Iijima S. (2009). Phys. Rev. Lett..

[cit13] Ullmann K., Coto P. B., Leitherer S., Molina-Ontoria A., Martin N., Thoss M., Weber H. B. (2015). Nano Lett..

[cit14] Nef C., Posa L., Makk P., Fu W., Halbritter A., Schonenberger C., Calame M. (2014). Nanoscale.

[cit15] Moser J., Bachtold A. (2009). Appl. Phys. Lett..

[cit16] Gehring P., Sadeghi H., Sangtarash S., Lau C. S., Liu J., Ardavan A., Warner J. H., Lambert C. J., Briggs G. A. D., Mol J. A. (2016). Nano Lett..

[cit17] Connolly M. R., Chiu K. L., Lombardo A., Fasoli A., Ferrari A. C., Anderson D., Jones G. A. C., Smith C.
G. (2011). Phys. Rev. B: Condens. Matter Mater. Phys..

[cit18] Shi S., Xu X., Ralph D. C., McEuen P. L. (2011). Nano Lett..

[cit19] Lu Y., Goldsmith B., Strachan D. R., Lim J. H., Luo Z., Johnson A. T. (2010). Small.

[cit20] Stampfer C., Schurtenberger E., Molitor F., Güttinger J., Ihn T., Ensslin K. (2008). Nano Lett..

[cit21] Barreiro A., Lazzeri M., Moser J., Mauri F., Bachtold A. (2009). Phys. Rev. Lett..

[cit22] Moser J., Barreiro A., Bachtold A. (2007). Appl. Phys. Lett..

[cit23] Ritter K. A., Lyding J. W. (2009). Nat. Mater..

[cit24] Wang X., Ouyang Y., Li X., Wang H., Guo J., Dai H. (2008). Phys. Rev. Lett..

[cit25] Yang L., Park C. H., Son Y. W., Cohen M. L., Louie S. G. (2007). Phys. Rev. Lett..

[cit26] Johnston D. E., Strachan D. R., Johnson A. T. C. (2007). Nano Lett..

[cit27] Warner J. H., Rummeli M. H., Ge L., Gemming T., Montanari B., Harrison N. M., Buchner B., Briggs G. A. D. (2009). Nat. Nanotechnol..

[cit28] Collins P. G., Hersam M., Arnold M., Martel R., Avouris P. (2001). Phys. Rev. Lett..

[cit29] Javey A., Guo J., Paulsson M., Wang Q., Mann D., Lundstrom M., Dai H. (2004). Phys. Rev. Lett..

[cit30] Sarwat S. G., Gehring P., Hernandez G. R., Warner J. H., Briggs G. A. D., Mol J. A., Bhaskaran H. (2017). Nano Lett..

