# Supplementary material for: Building nanogapped graphene electrode arrays by electroburning
Source: RSC Adv. 2018 Feb 12;8(13):6814–9. doi: 10.1039/c7ra13106b (PMC9078314; doi:10.1039/c7ra13106b)
Supplement: RA-008-C7RA13106B-s001 [file RA-008-C7RA13106B-s001.pdf]

Electronic Supplementary Material (ESI) for RSC Advances.
This journal is © The Royal Society of Chemistry 2018

Electronic Supplementary Information

Building Nanogapped Graphene Electrode Arrays by Electroburning

By Chunhui Gu[¶], *Dingkai Su*[¶], *Chuancheng Jia, Shizhao Ren, Xuefeng Guo**

[*] C. Gu, D. Su, Dr. C. Jia, S. Ren, Prof. Dr. X. Guo
Beijing National Laboratory for Molecular Sciences, State Key Laboratory for Structural Chemistry of Unstable and Stable Species, College of Chemistry and Molecular Engineering, Peking University, Beijing 100871, P. R. China.
Email: guoxf@pku.edu.cn

Prof. Dr. X. Guo
Department of Materials Science and Engineering, College of Engineering, Peking University, Beijing 100871, P. R. China.

[¶]These authors contributed equally to the work.

1. Device fabrication

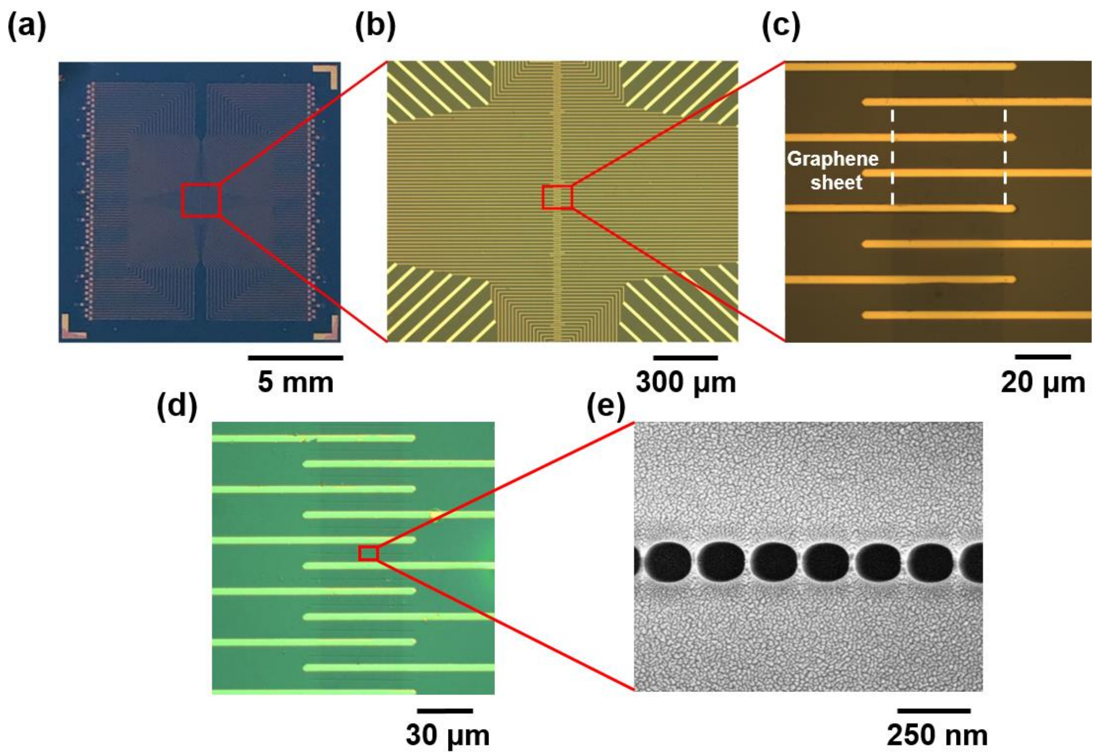

Figure S1. a-c) Optical images of a graphene device with different magnification. The yellow parts are metallic gold electrodes (Cr/Au: 8/60 nm), and the central part in (c) is graphene sheet (40 μm). d) Optical image of a graphene device coated by a PMMA layer after e-beam lithography. The center of each electrode pairs shows the exposed windows. e) SEM images of the exposed window arrays on PMMA resist (after gold spraying).

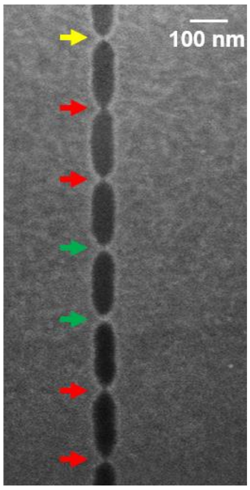

Figure S2. A typical SEM image of a graphene nanoconstriction array after oxygen RIE. The pattern units show inhomogeneity in some extent. The red, green and yellow arrows indicate for cracked units, uncracked units and units under the resolution, respectively.

2. Electrical measurement

The resistance of 40 μm wide graphene sheets (R_{ele}) and graphene nanoconstriction arrays (R_{nano}) were derived from the current measurement under a bias voltage U = 50 mV by an Agilent 4155C semiconductor characterization system (Figure S2a). Herein, R_{ele} equals U divided by the current before RIE. R_{ele} + R_{nano} equals U divided by the current after RIE.

Table S1: The resistance of the typical graphene devices before and after RIE etching (Region 1)

Device number	Current before RIE (μA)	Current after RIE (μA)	R_{ele}(kΩ)	R_{ele}+R_{nano}(kΩ)	R_{nano}(kΩ)	R_{ele}/R_{nano}
1	8.25	5.02	6.06	9.96	3.90	0.6
2	8.03	1.20	6.23	41.67	35.44	5.7
3	12.8	4.85	3.91	10.31	6.40	1.6
4	15.1	6.78	3.31	7.37	4.06	1.2
5	18.6	9.88	2.69	5.06	2.37	0.9
6	7.33	4.28	6.82	11.68	4.86	0.7
7	5.31	1.68	9.42	29.76	20.35	2.2
8	5.32	1.53	9.40	32.68	23.28	2.5
9	4.38	0.92	11.42	54.35	42.93	3.8
10	6.45	1.80	7.75	27.78	20.03	2.6
11	13.2	1.06	3.79	47.17	43.38	11.5
12	13.7	3.90	3.65	12.82	9.17	2.5
13	15.4	1.12	3.25	44.64	41.40	12.8
14	12.1	5.04	4.13	9.92	5.79	1.4
15	6.34	1.64	7.89	30.49	22.60	2.9
16	8.64	3.72	5.79	13.44	7.65	1.32
17	8.15	1.28	6.13	39.06	32.93	5.37
18	8.9	3.22	5.62	15.53	9.91	1.76
19	8.5	0.94	5.88	53.19	47.31	8.0
20	4.94	1.80	10.12	27.78	17.66	1.7
21	6.81	0.88	7.34	56.82	49.48	6.7
22	19.4	2.00	2.58	25.00	22.42	8.7
23	18.1	3.28	2.76	15.24	12.48	4.5
24	7.22	1.29	6.93	38.76	31.83	4.6
25	9.9	2.55	5.05	19.61	14.56	2.9
26	4.41	2.17	11.34	23.04	11.70	1.0
27	12.2	4.52	4.10	11.06	6.96	1.7
28	4.86	2.79	10.29	17.92	7.63	0.7
29	5.85	2.27	8.55	22.03	13.48	1.6
30	7.2	4.26	6.94	11.74	4.79	0.7

Table S2: The resistance of the typical graphene devices before and after RIE etching (Region 2)

Device number	Current before RIE (μA)	Current after RIE (μA)	R_{ele}(kΩ)	R_{ele}+R_{nano}(kΩ)	R_{nano}(kΩ)	R_{ele}/R_{nano}
1	13.4	0.064	3.73	781	778	208.4
2	14.7	0.02	3.40	2.50×10^3	2.49×10^3	734.0
3	15.6	0.50	3.21	100	97	30.2
4	5.05	0.04	9.90	1.25×10^3	1.24×10^3	125.3
5	5.32	0.27	9.40	185	176	18.7
6	4.49	0.10	11.14	500	489	43.9
7	6.6	0.02	7.58	2.50×10^3	2.49×10^3	329.0
8	9.12	0.045	5.48	1.11×10^3	1.10×10^3	201.7
9	8.07	0.13	6.20	385	378	61.1
10	8.83	0.067	5.66	746	741	130.8
11	8.48	0.016	5.90	3.13×10^3	3.12×10^3	529.0
12	8.04	0.385	6.22	130	124	19.9
13	6.95	0.32	7.19	156	149	20.7
14	7.94	0.14	6.30	357	351	55.7
15	6.87	0.10	7.28	500	493	67.7
16	6.42	0.068	7.79	735	728	93.4
17	5.73	0.12	8.73	417	408	46.8
18	5.72	0.57	8.74	88	79	9.0
19	7.11	0.30	7.03	167	160	22.7
20	5.26	0.003	9.51	1.67×10^5	2.49×10^5	1752.3
21	4.45	0.45	11.24	111	100	8.9
22	8.49	0.10	5.89	500	494	83.9
23	7.6	0.003	6.58	1.67×10^5	2.49×10^5	2532.3
24	7.24	0.045	6.91	1.11×10^3	1.10×10^3	159.9
25	5.77	0.005	8.67	1.00×10^5	1.00×10^5	1153.0
26	6.34	0.08	7.89	625	617	78.3
27	5.02	0.18	9.96	278	268	26.9
28	6.52	0.026	7.67	1.92×10^3	1.91×10^3	249.8
29	5.5	0.07	9.09	714	705	77.6
30	5.45	0.008	9.17	6.25×10^3	6.24×10^3	680.3
31	7.54	0.20	6.63	250	243	36.7
32	14.1	0.016	3.55	3.13×10^3	3.12×10^3	880.3
33	13	0.30	3.85	167	163	42.3
34	12.5	0.08	4.00	625	621	155.3
35	9.33	0.258	5.36	194	188	35.2

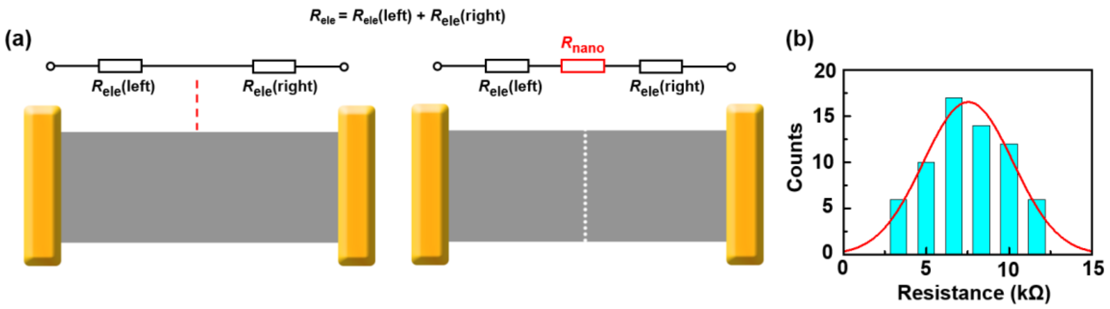

Figure S3. a) Schematic of the definition of R_{ele} and R_{nano}. b) Resistance distribution histogram of a 12 μm long, 40 μm wide graphene sheet. The resistance is fitted in 7.53 ± 2.42 kΩ, from which the resistivity ρ of single-layer graphene we calculated is 25.1 ± 8.1 kΩ.

3. The simulation based on a classical resistance model.

The relationship between the crack critical point (I, U) and the width of each graphene nanoconstriction unit (W_{unit}) was calculated based on an ideal broadening model (Figure 1b in the main text). The simulation was carried out by a numerical calculation method according to the following equation set with a Matlab R2009a software.

$$Part(x) = \frac{d_2 - 2 \times \sqrt{r^2 - x^2}}{d_1 + d_2}$$

$$R_{nano} = \int_{-r}^{r} \frac{(\rho/W_{chan})dx}{Part(x)}$$

$$R_{ele} = (\rho/W_{chan})(L_{chan} - 2r)$$

$$I = Jh(d_1 - 2r)n_{jun}$$

$$U = I\,(R_{nano} + R_{ele})$$

$$W_{unit} = d_2 - 2r$$

In the equation set, $Part(x)$ represents the remaining proportion of graphene on a particular line vertical to the channel. d_1 and d_2 represent the length of alternating segments (d_1) and spaces (d_2) in the dash line pattern. r represents the broadening radius, ρ represents the 2D resistivity for graphene, W_{chan} and L_{chan} represent the width and length for the graphene channel. R_{nano} and R_{ele} represent the resistance of the nanoconstriction array and graphene electrodes. J represents the critical current intensity and h represents the thickness of single-layer graphene.

In the numerical calculation, r was set as an independent variable from 15 nm to 20 nm. The following parameters were derived from the device design: d_1 = 150 nm, d_2 = 40 nm, W_{chan} = 40 μm, L_{chan} = 12 μm. ρ = 25.1 ± 1.7 kΩ was derived from the experimental results (Figure S2b). J = ~10^8 A/cm^2 and Jh = ~1 μA/nm were derived from the results reported before.

Equations E2 and E3 were derived from the classical Ohm's law: $I = U/R$, where I represents the current, U represents the applied voltage and R represents the resistance of the circuit. In E2, R is composed of the resistance of graphene sheets (R_{ele}) and graphene nanoconstriction arrays (R_{nano}): $R = R_{ele} + R_{nano}$ (see Figure S3). R_{nano} can be deemed as the resistance of graphene nanoconstriction arrays with n−paralleled units: $R_{nano} = R_{unit}$/n, where R_{unit} represents the resistance of each unit. In E3, the positive feedback ratio can be regarded as the normalized total differential: $\mathrm{d}I/I\mathrm{d}n$, evaluating how much proportion of the current ($\mathrm{d}I/I$) can be affected by the paralleled numbers ($\mathrm{d}n$).

4. The *I-V* curves of the electroburning process

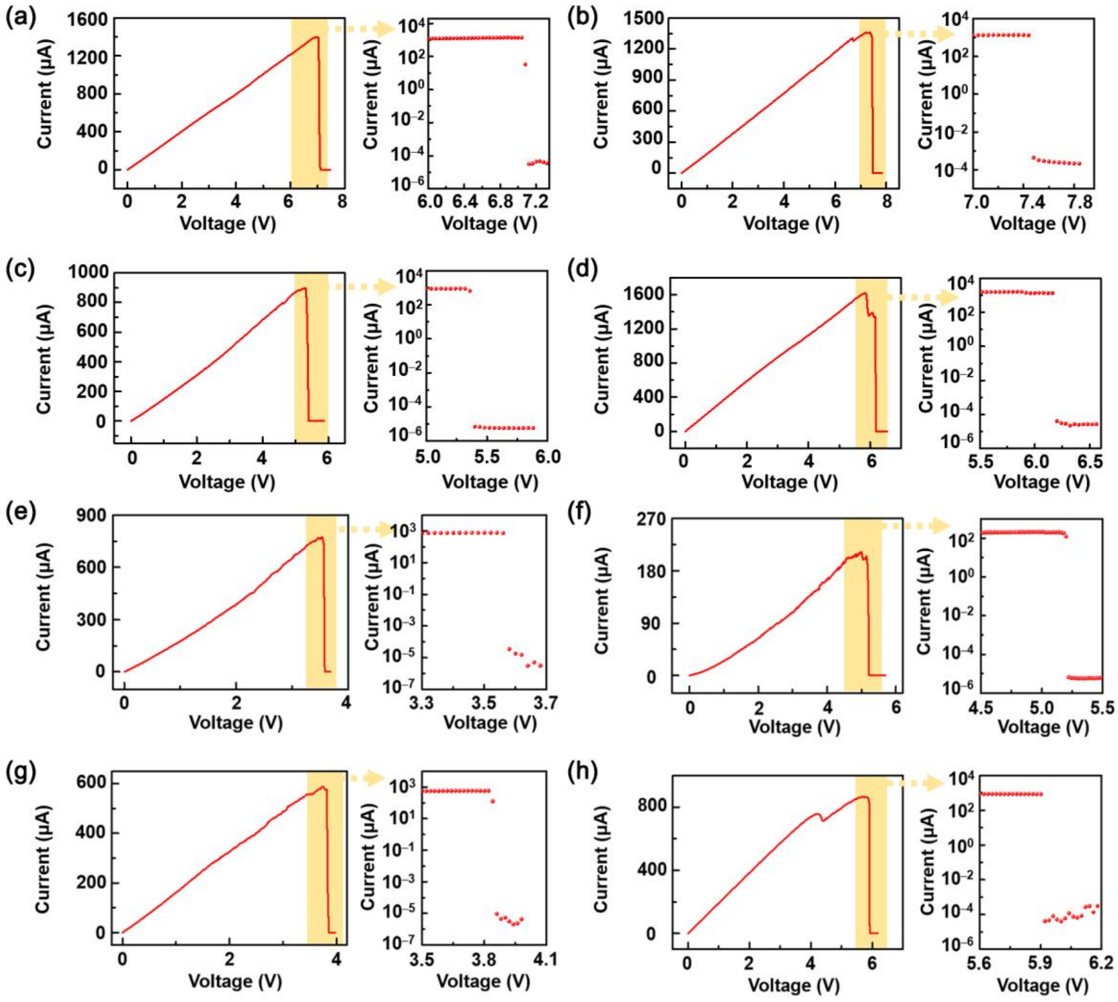

Figure S4. a-h) Representative *I-V* characteristics of the electroburning process, where the crack critical points are located in Region 1. Right figures show the enlarged scales of the yellow area in a semi-logarithm axis.

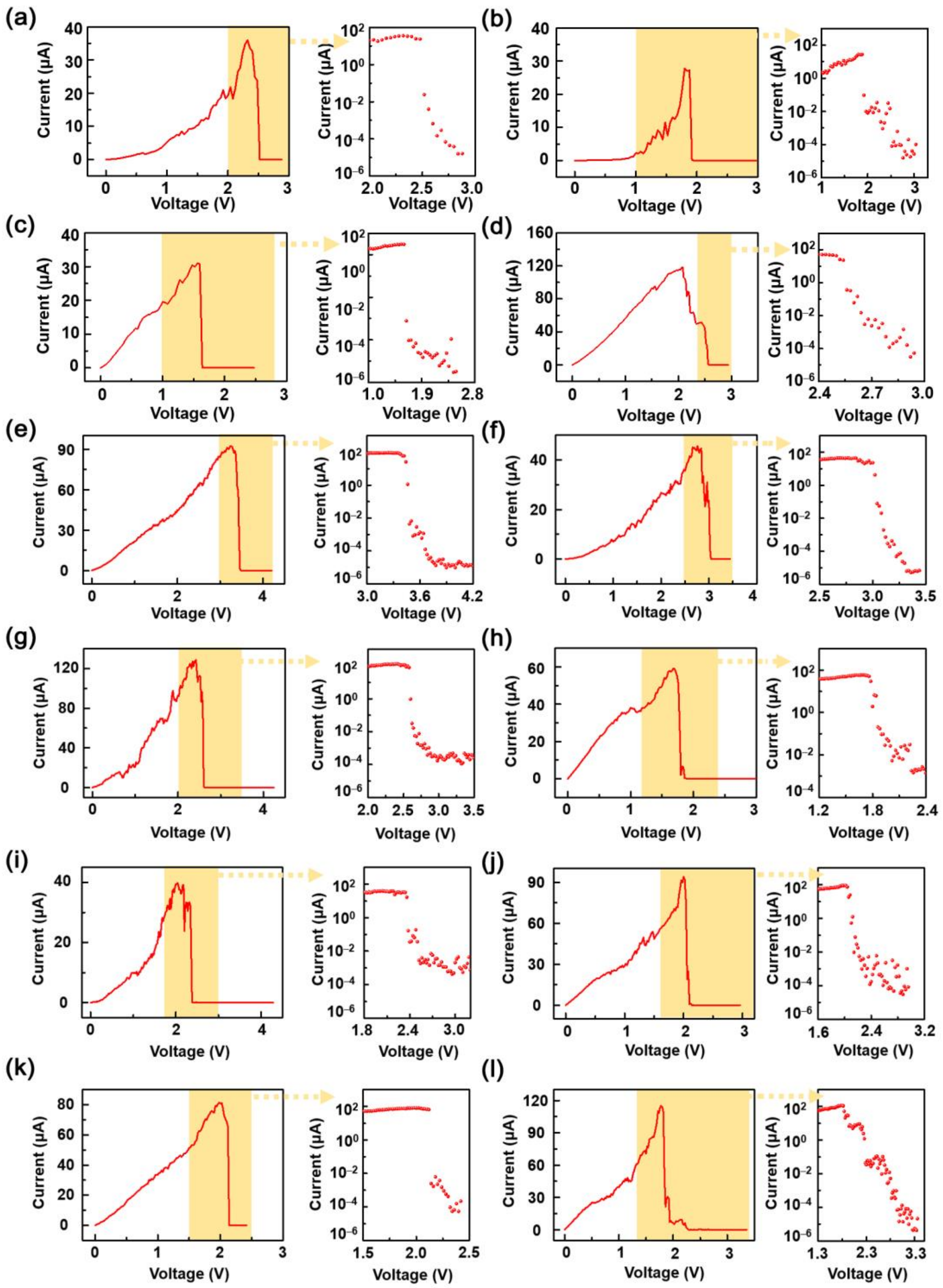

Figure S5. a-l) Representative *I-V* characteristics of the electroburning process, where the crack critical points are located in Region 2. Right figures show the enlarged scales of the yellow area in a semi-logarithm axis.

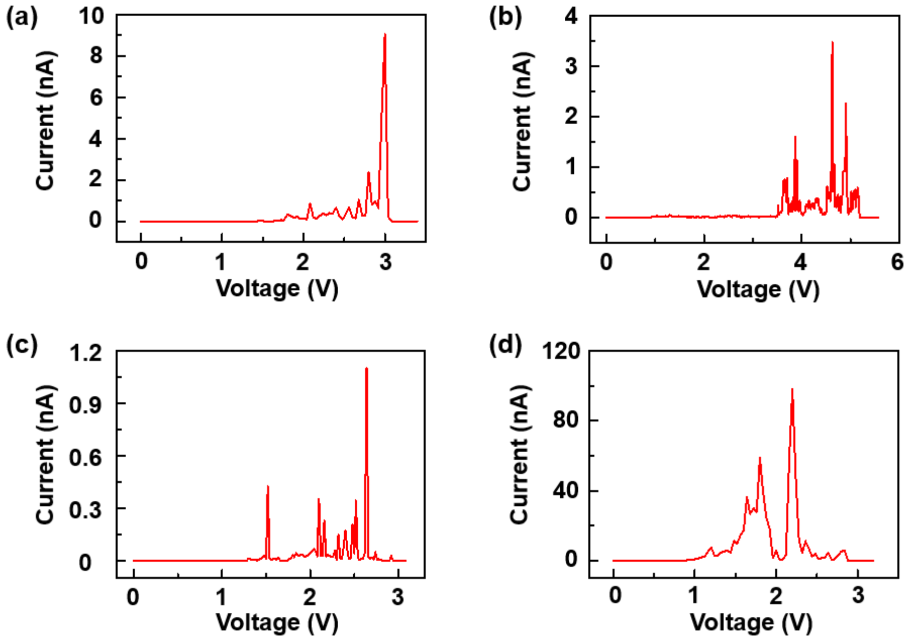

Figure S6. a-d) Representative *I-V* characteristics of the devices after the electroburning process, where the crack critical points are located under Region 2.
